# STRATEGIES FOR CONNECTING AGE INCLUSIVITY TO DEI EFFORTS: A CAMPUS CASE STUDY

**DOI:** 10.1093/geroni/igac059.1064

**Published:** 2022-12-20

**Authors:** Carrie Andreoletti, Andrea June

**Affiliations:** Central Connecticut State University, New Britain, Connecticut, United States; Central Connecticut State University, New Britain, Connecticut, United States

## Abstract

We’ve employed a multi-pronged approach to connecting our AFU initiatives, which promote age inclusivity, to our university’s DEI efforts. First, we asked our DEI office if they would collaborate. They agreed to link our AFU webpage to the DEI webpage and supported Ageism First Aid training for faculty and staff. Second, we participated in equity, justice, and inclusion (EJI) efforts on campus by ensuring that gerontology courses qualified for the EJI designation. This helps expand aging education across the curriculum as all students must take one EJI designated course. We also volunteered to speak about ageism to the first cohort of John Lewis Institute Scholars. Third, we partnered with our Center for Teaching and Innovation to offer programming on age inclusivity and generational diversity in the classroom. Taken together, these efforts have helped us to expand our reach and ensure that age is part of DEI conversations on our campus.

